# Dengue virus serotype distribution based on serological evidence in pediatric urban population in Indonesia

**DOI:** 10.1371/journal.pntd.0006616

**Published:** 2018-06-28

**Authors:** R. Tedjo Sasmono, Anne-Frieda Taurel, Ari Prayitno, Hermin Sitompul, Benediktus Yohan, Rahma F. Hayati, Alain Bouckenooghe, Sri Rezeki Hadinegoro, Joshua Nealon

**Affiliations:** 1 Eijkman Institute for Molecular Biology, Jakarta, Indonesia; 2 Sanofi Pasteur, Asia & JPAC Region, Singapore; 3 Department of Child Health, Faculty of Medicine, Universitas Indonesia, Cipto Mangunkusumo Hospital, Jakarta, Indonesia; Fundacao Oswaldo Cruz, BRAZIL

## Abstract

**Background:**

Dengue is a febrile illness transmitted by mosquitoes, causing disease across the tropical and sub-tropical world. Antibody prevalence data and serotype distributions describe population-level risk and inform public health decision-making.

**Methodology/Principal findings:**

In this cross-sectional study we used data from a pediatric dengue seroprevalence study to describe historical dengue serotype circulation, according to age and geographic location. A sub-sample of 780 dengue IgG-positive sera, collected from 30 sites across urban Indonesia in 2014, were tested by the plaque reduction neutralization test (PRNT) to measure the prevalence and concentration of serotype-specific neutralizing antibodies according to subject age and geography. PRNT results were obtained from 776 subjects with mean age of 9.6 years. 765 (98.6%) neutralized one or more dengue serotype at a threshold of >10 (1/dil). Multitypic profiles were observed in 50.9% of the samples; a proportion which increased to 63.1% in subjects aged 15–18 years. Amongst monotypic samples, the highest proportion was reactive against DENV-2, followed by DENV-1, and DENV-3, with some variation across the country. DENV-4 was the least common serotype. The highest anti-dengue antibody titers were recorded against DENV-2, and increased with age to a geometric mean of 516.5 [1/dil] in the oldest age group.

**Conclusions/Significance:**

We found that all four dengue serotypes have been widely circulating in most of urban Indonesia, and more than half of children had already been exposed to >1 dengue serotype, demonstrating intense transmission often associated with more severe clinical episodes. These data will help inform policymakers and highlight the importance of dengue surveillance, prevention and control.

## Author summary

Dengue is a febrile illness transmitted by mosquitoes, causing disease across the tropical and sub-tropical world. Antibody prevalence data and serotype distribution describe population-level risk and inform public health decision-making. We present data from a dengue seroprevalence study in children in Indonesia; circulation of the four dengue serotypes (DENV-1, -2, -3, -4) was assessed, by age and location. Samples collected from 30 urban Indonesian sites were tested using the plaque reduction neutralization test (PRNT), which enabled us to measure prevalence and concentration of antibodies specific to dengue virus serotypes. Results were obtained from 776 subjects (mean age: 9.6 years). 765 (98.6%) neutralized ≥1 dengue serotype; the highest proportion was reactive against DENV-2, followed by DENV-1, and DENV-3, with some variation across the country. Reaction to multiple serotypes was observed in 50.9% of samples. The highest anti-dengue antibody titers were recorded against DENV-2, and increased with age. The fact that all four dengue serotypes have been widely circulating in urban Indonesia, and more than half of children had been exposed to >1 dengue serotype, shows intense transmission, often associated with more severe clinical episodes. These data will help inform policymakers and highlight the importance of dengue surveillance, prevention and control.

## Introduction

Dengue is a febrile illness caused by dengue virus (DENV) infection. The clinical manifestations of dengue occur on a spectrum, ranging from asymptomatic or a mild flu-like syndrome known as classic dengue fever (DF), to a more severe form known as dengue hemorrhagic fever (DHF) and the potentially fatal dengue shock syndrome (DSS) [1]. DENV, which belongs to the family *Flaviviridae*, is transmitted by mosquitoes of the genus *Aedes*; predominantly *Aedes aegypti*. There are four evolutionarily distinct, antigenically related DENV serotypes; DENV-1, -2, -3, and -4 causing disease across the tropical and sub-tropical world [2, 3].

Neutralizing antibodies (NAbs) against the four serotypes are considered a critical component of the protective immune response which is achieved when adequate, specific antibody titers circulate [4]. Accordingly, plaque reduction neutralization tests (PRNT), which quantify serum concentrations required to neutralize live viruses, are the most specific assays for detecting flavivirus exposure history [5]. The dengue PRNT is able to target individual viral serotypes, and therefore can infer serotype-exposure history, however, interpretation of heterotypic responses is complicated for reasons including original antigenic sin [6, 7].

Indonesia is the largest archipelago country in the world with over 17,000 islands, inhabited by around 240 million people. Dengue was first reported in 1968, and has been expanding ever since, in both incidence and geography, with an annual burden of >750,000 cases [8]. The disease is likely hyperendemic across most islands [9, 10]. Reporting of DHF in Indonesia is mandatory within 72 hours of diagnosis, health centers and public/private hospitals use the World Health Organization’s (WHO) 1997 case definitions [11] and only DHF/DSS cases are reported. Laboratory confirmation of dengue is rare, especially in health services with limited facilities although dengue IgG/IgM and NS1 rapid tests are increasingly used in hospitals and health clinics. Indonesia does not conduct nationally-representative dengue serotype surveillance. Genotypic and serological surveillance has been undertaken by some Indonesian institutions, on a project basis which confirmed the dengue serotypes in symptomatic individuals [12–14]. Those studies include in Makassar, South Sulawesi from 2007–2010, where dengue infection was confirmed in >100 patients, many of whom were aged 11–20 years old. Serotyping revealed that DENV-1 was the most common form (41%) followed by DENV-2 (31%), DENV-3 (20%), and DENV-4 (7%) [15]. In Surabaya, East Java, in 2012, dengue RNA was isolated from 79 of 148 suspected dengue patients (53%), with DENV-1 as the predominant serotype (73%), followed by DENV-2 (8%), DENV-4 (8%), and DENV-3 (6%), while 5% were found to have mixed serotypes [16]. In Semarang, Central Java in 2012, 66 of 120 suspected cases (55%) were serologically confirmed and viral RNA was detected in 31 samples [12]. DENV-1 was the predominant serotype, followed by DENV-2, DENV-3, and DENV-4. DENV-1 predominance has also been reported from other studies and cities in Indonesia, including Surabaya [17] and Makassar [15]. Finally, from urban and rural areas of Bali (Denpasar and Gianyar), in 2015, 205 adult patients with suspected dengue were recruited in a prospective cross-sectional study. Of these, 161 patients had virologically-confirmed dengue; DENV-3 was predominant (48%), followed by DENV-1 (28%), DENV-2 (17%), and DENV-4 (4%). Five samples (3%) were detected which contained two different serotypes, and it was noted that the proportions varied in urban and rural areas [18].

Understanding antibody prevalence is an important consideration in the interpretation of epidemiological data, especially when reviewing interactions with other flaviviruses or considering vaccine introduction. The co-circulation of multiple dengue serotypes is a population-level risk factor for severe dengue disease because of the increased likelihood of a second or subsequent infection, and also due to the fact that sequential infections are associated with increased severity [19]. Serotype distribution may be predictive of future epidemiology and is important information for dynamic transmission models. The objective of this study was to use data from a dengue seroprevalence survey to describe the historical serotype (DENV-1, 2, 3, 4) circulation based on the prevalence of serotype-specific anti-DENV antibodies, according to age and geographic location, in a pediatric population in Indonesia.

## Materials and methods

### Study design

In this cross-sectional study, serum samples and data from a national-level pediatric dengue seroprevalence study were used to describe historical dengue serotype circulation, according to age and geographic location. Dengue IgG-positive sera, collected from 30 sites across urban Indonesia, were tested by the PRNT to measure the prevalence and concentration of serotype-specific dengue neutralizing antibodies according to subject age and geography.

### Sample collection and selection

Surveillance and sample collection methods were previously described [20]. Ethical approval was obtained from the Health Research Ethics Committee of Faculty of Medicine of University of Indonesia (No. 462/H2.F1/ETIK/2014). Briefly, between 30 October 2014–27 November 2014, blood samples were collected from 3,210 children aged 1–18 years in 30 urban Indonesian subdistricts, randomly selected from west to east based on the probability proportional to population size. The blood samples were to be tested for dengue IgG by enzyme-linked immunosorbent assay (ELISA). A sub-sample of 780 dengue IgG positive sera was used to estimate the prevalence of serotype-specific neutralizing antibodies by PRNT. The sample size was estimated to provide 95% confidence and a margin error of 5%; this is accounting for the 30 clusters with a design effect of two and assuming the “worst case” of 50% exposure to any one serotype. The sample was not strictly representative of the dengue IgG positive population as the samples were selected equally from each of the four age groups, *i*.*e*. 195 samples per age group, and, to provide geographical representativeness, from clusters in proportion to dengue IgG seroprevalence rates. This method over-sampled from younger subjects to; 1) increase the number of samples tested from children recently infected with dengue, to provide a record of recent dengue circulation; 2) reduce the number of PRNTs performed on samples from older children, likely to have been infected with many serotypes, which may therefore be impossible to meaningfully interpret.

### Dengue plaque reduction neutralization test (PRNT)

The PRNT method was performed based on optimized and validated PRNT_50_ assay for the detection of neutralizing antibodies to four serotypes of DENV [21]. Each serum sample was heat inactivated at 56°C and assayed in four separate PRNT runs, which corresponded to four different DENV serotype challenge viruses. Vero cells (CCL-81) were obtained from American Type Culture Collection (ATCC). Cells were grown and maintained in Minimum Essential Medium (MEM) (Gibco-Thermo Fisher Scientific, CA, USA), supplemented with 5% heat-inactivated Fetal Bovine Serum (FBS), 2 mM of L-glutamine, and 1% of antibiotic/antimycotic (Gibco-Thermo Fisher Scientific, CA, USA) at 37°C in an atmosphere of 5% CO_2_. Working banks of Vero cells were prepared in-house, qualified, and confirmed to be free of any microbial, mycoplasma, and viral contaminants. Purified mouse monoclonal antibodies (MAbs) specific to the DENV serotype envelope protein were used as the primary antibodies for virus detection according to the corresponding serotype: anti-DENV-1 (D2-1F1-3), anti-DENV-2 (3H5-1-12), anti-DENV-3 (8A1-2F12), and anti-DENV-4 (1H10-6-7) (Biotem, Le Rivier d’Apprieu, France). Alkaline phosphatase-conjugated goat anti-mouse IgG (Jackson Immunoresearch Laboratories, West Grove, PA, USA) was used as the secondary antibody. The parental DENVs of the recombinant CYD vaccine viruses, i.e., DENV-1 strain PUO-359, DENV-2 strain PUO-218, DENV-3 strain PaH881/88, and DENV-4 strain 1228, were used as challenge viruses in the PRNT. The initial source, and the suitability of these four DENV serotypes to be used in dengue neutralization assay have been described elsewhere [21–23]. Dengue-antibody positive and negative human serum sample controls were obtained from healthy adult donors from Indonesia. The serum controls were used in each assay run, and served to monitor its performance and validity.

The neutralization titer (PRNT_50_) of the test serum sample was defined as the reciprocal of the highest test serum dilution for which the virus infectivity was reduced by 50% when compared with the average plaque count of the challenge virus control, calculated using a four-point linear regression method. Since the lowest starting dilution of serum in the assay was 1:5, the theoretical lower limit of quantitation of the assay was a titer of 10 (reciprocal dilution).

### Statistical analysis

This is a descriptive analysis, no hypotheses were tested. The study population mean age was calculated and geographic distribution described. Dengue serotype specific PRNT profiles were defined according to the following algorithm; categorizing samples as naïve (no previous dengue infection), monotypic (infection with one serotype), or multitypic (>1 serotype)[24]:

- Naïve: antibody titers <10 for the four serotypes
- Monotypic: antibody titers >10 (1/dil) to only one serotype **or** titers ≥ 10 for different serotypes with a high titer (>80 (1/dil)) and for a single predominant serotype (> 5 times higher than other titers)
- Multitypic: antibody titers ≥10 (1/dil) for different serotypes without a single predominant titer.

PRNT profile distribution by age and geography were described. PRNT profile prevalence and their 95% confidence intervals (95% CI) were calculated, the clusters results were aggregated at province level and a map was generated using QGIS 2.16.2 “Nødebo”.

The mean PRNT titer, GMT (Geometric Mean Titer), and the 95% CI for each age group and dengue serotype was calculated for all samples based on their DENV PRNT results. To calculate the GMT, samples with an antibody titer T <10 (1/dil) were given the value 5 and the mean titer was calculated using the equation:

GMT^jh=10^∑1nlog10Tin

Where ${\hat{GMT}}_{jh}$ is the mean titer for the dengue serotype *h* of the age group *j*, *T*_*i*_ is the PRNT titer of the subjects *i* and *n* the number of subjects with a PRNT titer in the age group *j* for the serotype *h*.

All statistical analyses were performed using Excel 2013.

## Results

### Description of sample set

Blood samples were collected from 3,210 children aged 1–18 years in 30 urban Indonesian subdistricts, randomly selected from west to east. From a sub-sample of 780 dengue IgG positive sera, PRNT_50_ results were obtained from 776 participants, equally sampled from each age group (1–4, 5–9, 10–14 and 15–18 years old). In the youngest, 1–4 years old group, four serum samples were of insufficient quantity to be tested. The mean age was 9.6 years old (95% CI [9.3–10.0]. The 30 clusters were represented with 14–39 samples per cluster. Of these, 765 (98.6%) neutralized one or more dengue serotypes at a threshold of >10 (1/dil), a proportion which varied by age: 95.3% in the 1–4 years old, 99.5% in the 5–9 years old, 99.5% in the 10–14 years old and 100% in the 15–18 years old.

### PRNT profile distribution stratified by age and geographic level

**Fig 1:**
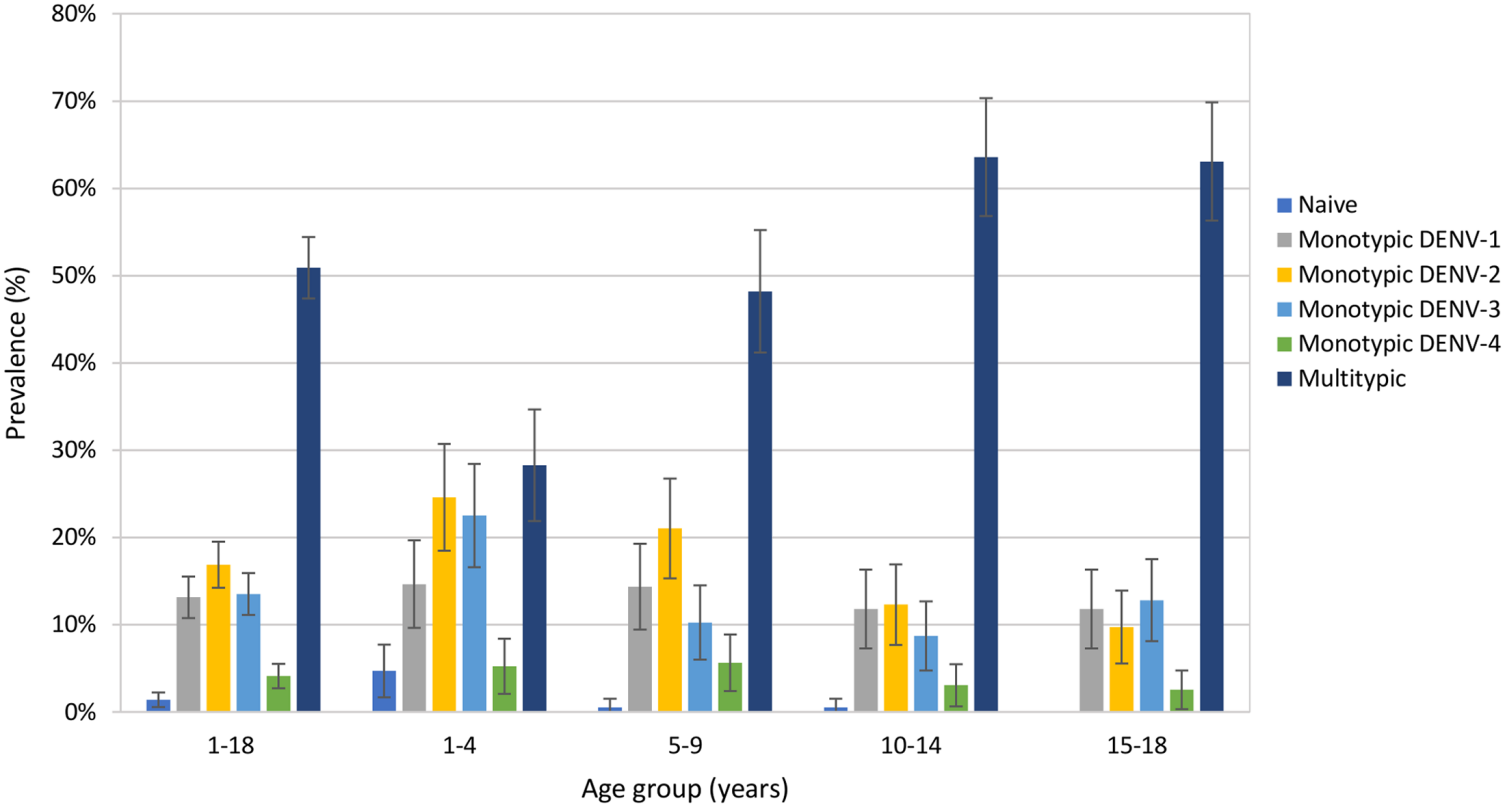
Proportion of individuals with naïve, monotypic (for each dengue serotype), or multitypic PRNT profiles, by age group.

Samples were categorized according to PRNT_50_ profile. Multitypic profiles were observed in 50.9% of the subjects, with 28.3% in those aged 1–4 years old, 48.2% in the 5–9 years old, 63.6% in the 10–14 years old and 63.1% in those aged 15–18 (Fig 1). The proportion of monotypic profiles decreased with increasing age, representing 67.0% of those aged 1–4 years, 51.3% of the 5–9 year old group, 35.9% of the 10–14 years old group, 36.9% of the 15–18 years old and 47.7% of the overall sample. There were no naïve subjects in the 15–18 years old group whereas 4.7% of the 1–4 years old group; 0.5% of the 1–9 and 10–14 years old groups, and 1.4% of the overall sample had no detectable neutralizing dengue antibodies at the 10 (1/dil) threshold. Amongst monotypic samples, the highest proportion of samples were reactive against DENV-2, followed by DENV-1, and DENV-3, a trend which was also observed in the two youngest age groups, while the three serotypes were more evenly distributed amongst the 10–14 and 15–18 years old age groups (Fig 1).

**Fig 2:**
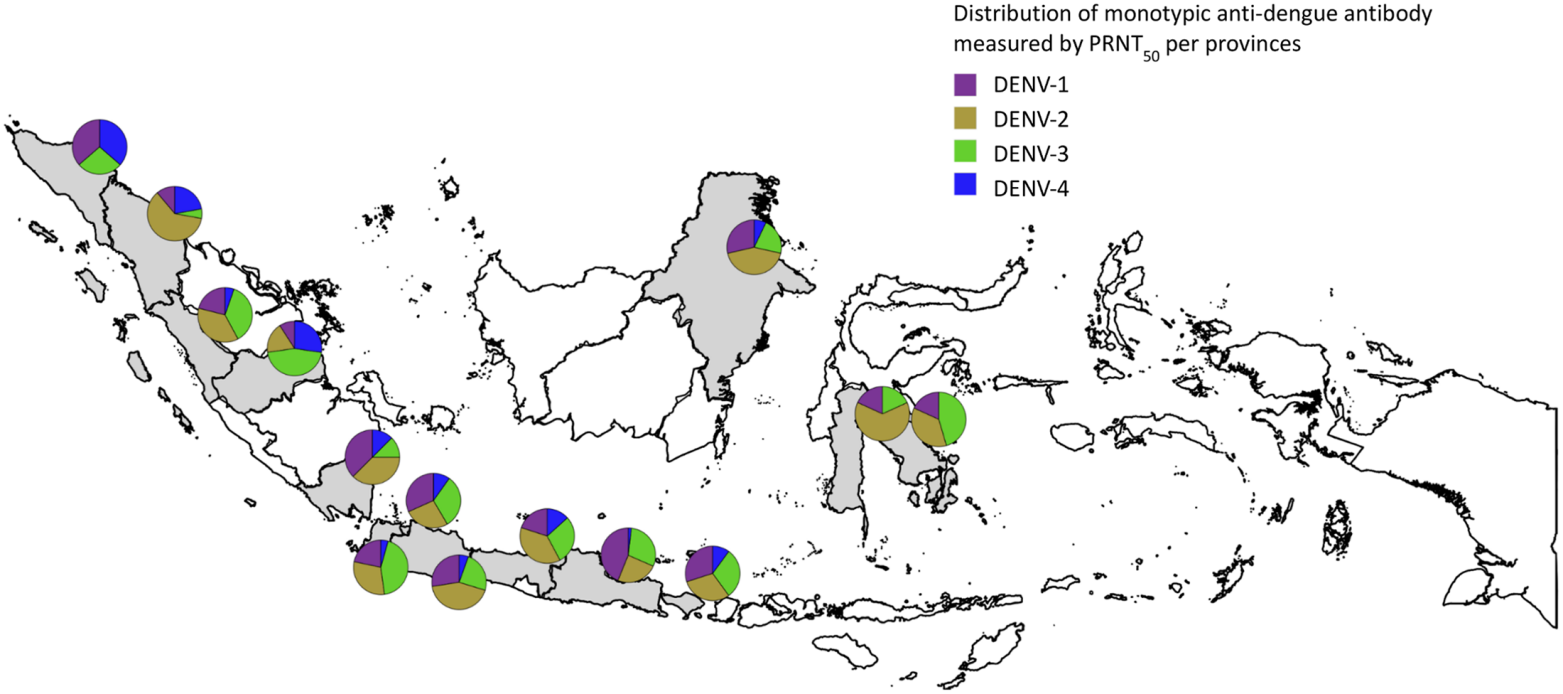
Map showing the proportion of monotypic dengue antibody profiles against each dengue serotype, by Indonesian province containing at least one study site (shown in grey).

The clusters were aggregated within 14 provinces, resulting in samples per province ranging from 15 to 183 serum samples. In seven provinces multitypic profiles were the most common (from 52.2% to 69.4% of samples). In seven provinces the monotypic profile was more prevalent (from 49.7% to 68.8%). DENV-4 was dominant in one province, in the 13 other provinces DENV-1, DENV-2, DENV-3 or a combination of these serotypes were dominant, with DENV-2 dominance being more common (Fig 2). The four monotypic serotypes were identified in every province, with the exception of DENV-2 in Nanggroe Aceh Darussalam and DENV-4 in Sulawesi Tenggara and Sulawesi Selatan.

### GMT stratified by age

**Fig 3:**
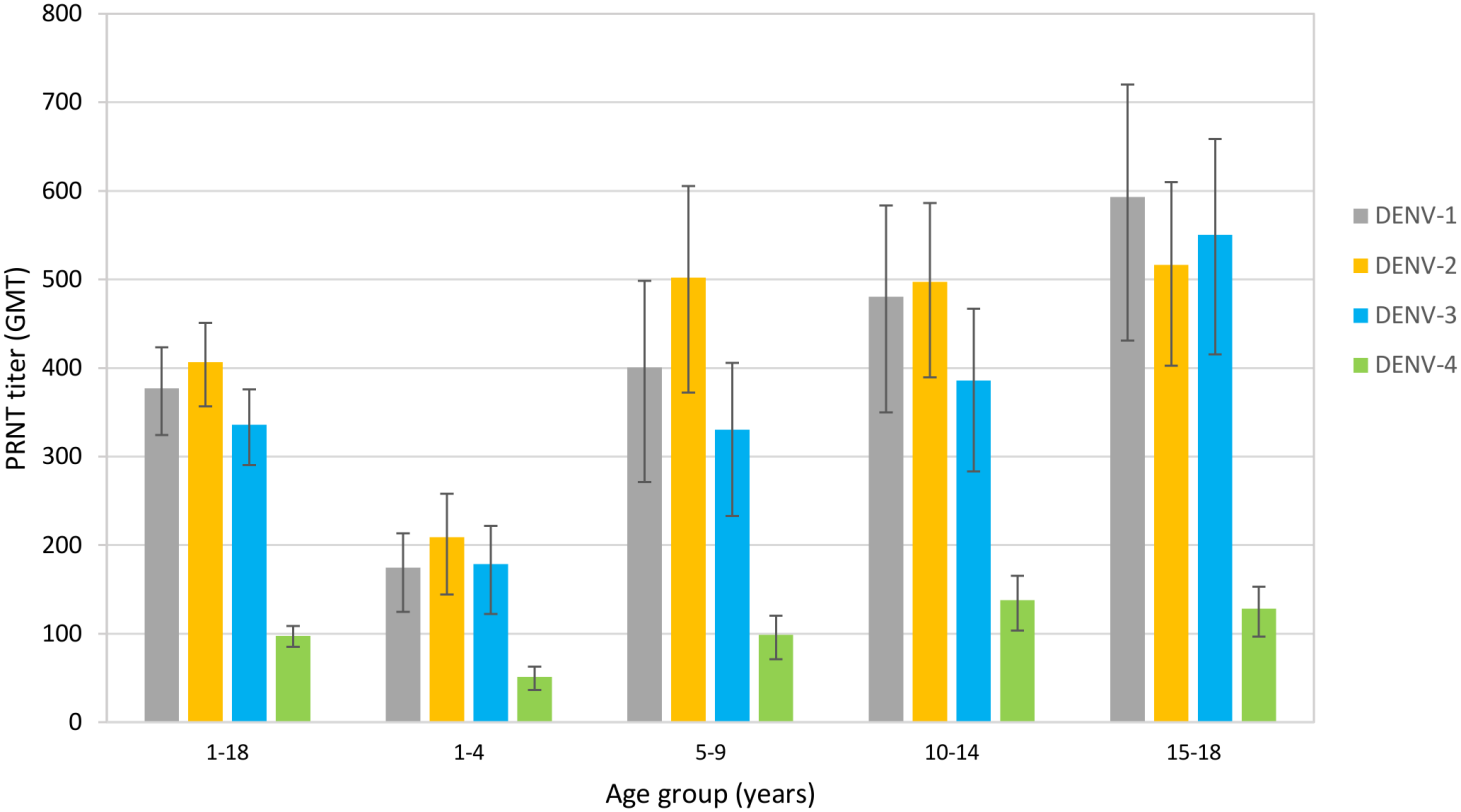
Dengue serotype specific GMT by age-group.

GMTs increased with age. DENV-2 had the highest GMT overall (406.5 [1/dil]) and for three of the four age groups with titers of 208.8, 502.2, 497.4 and 516.5 [1/dil], respectively (Fig 3). DENV-4 had the lowest GMT for each age group (51.2, 98.9, 138.1 and 128.2 [1/dil]) and overall (97.6 [1/dil]). In the oldest subjects, titers against DENV-1 were highest (593.08 [1/dil]) followed by DENV-3 (550.2 [1/dil]) and DENV-2.

## Discussion

We conducted a dengue seroprevalence study which identified serological evidence for the circulation of all four dengue serotypes across urban areas of Indonesia, in children who were exposed to infection from 1996 to 2013. The proportion of children with exposure to >1 serotype increased with age, and children were more likely to have been infected with DENV-2, DENV-1 and DENV-3 than DENV-4. Nonetheless, these results show that all four serotypes have been widely circulating in most of Indonesia, as is common in hyper-endemic countries. This study generated data on serotype-specific prevalence in areas where little or no data were previously available, with the exception of historical data from Yogyakarta, Java island [32].

Available dengue serotype data collated from 1994 to 2012 (n = 596) [25] and recent publications from all over Indonesia confirm the concomitant presence of the four DENV serotypes [10, 12, 15–18, 26–28]. Samples were collected from suspected cases and therefore suffer a potential selection bias towards serotypes associated with more symptomatic/severe cases. The serological data we report here indicate a consistent pattern of distribution of serotypes, a finding which may indicate that the cases captured within these surveillance studies is broadly reflective of the DENV serotype circulation in the country.

PRNT enables the interrogation of samples according to their exposure history. In this study, it was remarkable to observe that in this pediatric population more than half (50.9%) had already been exposed to >1 dengue serotype, a proportion which increased with age. This rate is important because it demonstrates early and intense transmission in Indonesia; and we know that second infections have been described as more likely to be symptomatic, severe and hemorrhagic [29]. Individuals of an age likely to have received one natural exposure, but before their second, may represent an attractive target for dengue vaccination programs [30]. The observed GMT increase with age is most likely explained by continuous re-exposure to DENVs over time, further boosting antibody levels. These profiles imply that existing vector control activities in urban areas are largely insufficient at preventing infection; and that investments in novel methods may be warranted. The prevalence of multitypic profiles further reinforces the requirement for development of a safe and effective, quadrivalent dengue vaccine which could be used in children at highest risk of developing symptomatic and severe disease episodes. Additionally, these data can be useful for the calibration of dengue transmission models which may help to understand disease dynamics and the likely effects of dengue control interventions.

There are several limitations to our study. Sera collected during the convalescent phase represent infection history in the population, but are limited by the sensitivity and specificity of the serological methods used to quantify antibodies. We had the benefit of analyzing samples in this study by PRNT; however interpretation of data can be confused by heterotypic cross-neutralization between serotypes. For this reason, we did not interpret the serotype distributions of multitypic infections. Only samples positive for dengue IgG in ELISA screening assay were selected to undergo PRNT, therefore these may not be fully representative of dengue positive sera. We also observed discrepancies between IgG ELISA and PRNT data in which some samples that were positive by IgG ELISA were negative in PRNT (1.4%). This may be a consequence of the well-documented serological cross-reactivity across the flavivirus group [31] Our sample collection was also limited to urban areas and subjects consenting to the study which may have introduced additional bias.

In conclusion, this study confirmed the distribution of multiple dengue serotypes across urban Indonesia. Many children were infected with multiple serotypes, and the accompanying risk of severe disease, from an early age. DENV-1, DENV-2 and DENV-3 may play a more significant epidemiological role than DENV-4. It is hoped that these data influence policymakers to afford increased attention to dengue surveillance, prevention and control.

## Supporting information

S1 Checklist STROBE checklist. (DOC)

S1 Table Description of mean age, sample size and dengue serotype specific prevalence (naïve, monotypic, or multitypic) per province. (DOCX)

S2 Table IgG seroprevalence distribution per cluster and age group and derived PRNT sample size. (DOCX)
