# Supplementary material for: Dengue virus serotype distribution based on serological evidence in pediatric urban population in Indonesia
Source: PLoS Negl Trop Dis. 2018 Jun 28;12(6):e0006616. doi: 10.1371/journal.pntd.0006616 (PMC6040755; doi:10.1371/journal.pntd.0006616)
Supplement: S1 Checklist — (DOC) [file pntd.0006616.s001.doc]

STROBE Statement—Checklist of items that should be included in reports of ***cross-sectional studies***

|  | Item No | Recommendation | Location in manuscript |
| --- | --- | --- | --- |
| **Title and abstract** | 1 | (*a*) Indicate the study’s design with a commonly used term in the title or the abstract  *In this cross-sectional study we used data from a pediatric dengue seroprevalence study to describe historical dengue serotype circulation, according to age and geographic location, in a pediatric Indonesian population.* | Abstract – methodology |
|  |  | (*b*) Provide in the abstract an informative and balanced summary of what was done and what was found  *We present data from a dengue seroprevalence study in children in Indonesia; circulation of the four dengue serotypes (DENV-1, -2, -3, -4) was assessed, by age and location.* *Results were obtained from 776 subjects (mean age: 9.6 years). 765 (98.6%) neutralized ≥1 dengue serotype; the highest proportion was reactive against DENV-2, followed by DENV-1, and DENV-3, with some variation across the country. Reaction to multiple serotypes was observed in 50.9% of samples.* | Abstract and author summary |
| Introduction | | |  |
| Background/rationale | 2 | Explain the scientific background and rationale for the investigation being reported  *Dengue is a febrile illness caused by dengue virus (DENV) infection. The clinical manifestations of dengue occur on a spectrum, ranging from asymptomatic or a mild flu-like syndrome known as classic dengue fever (DF), to a more severe form known as dengue hemorrhagic fever (DHF) and the potentially fatal dengue shock syndrome (DSS). DENV, which belongs to the family Flaviviridae, is transmitted by mosquitoes of the genus Aedes; predominantly Aedes aegypti. There are four evolutionarily distinct, antigenically related DENV serotypes; DENV-1, -2, -3, and -4 causing disease across the tropical and sub-tropical world.*  *Neutralizing antibodies (NAbs) against the four serotypes are considered a critical component of the protective immune response which is achieved when adequate, specific antibody titers circulate. Accordingly, plaque reduction neutralization tests (PRNT), which quantify serum concentrations required to neutralize live viruses, are the most specific assays for detecting flavivirus exposure history. The dengue PRNT is able to target individual viral serotypes, and therefore can infer serotype-exposure history, however, interpretation becomes complicated following subsequent infections, for reasons including original antigenic sin.* | Introduction, paragraphs 1 and 2 |
| Objectives | 3 | State specific objectives, including any prespecified hypotheses  *Understanding antibody prevalence is an important consideration in the interpretation of epidemiological data, especially when reviewing interactions with other flaviviruses or considering vaccine introduction. The co-circulation of multiple dengue serotypes is a population-level risk factor for severe dengue disease, this is because of the increased likelihood of a second or subsequent infection, and also due to the fact that sequential infections are associated with increased severity. Serotype distribution may be predictive of future epidemiology and is important information for dynamic transmission models. Here, we use data derived from a pediatric dengue seroprevalence study to describe the dengue serotype (DENV-1, 2, 3, 4) specific circulation. This is based on the presence of anti-DENV antibodies, according to age and geographic location, in a pediatric population in Indonesia.* | Introduction, paragraph 4 |
| Methods | | |  |
| Study design | 4 | Present key elements of study design early in the paper  *In this cross-sectional study, data from a pediatric dengue seroprevalence study was used to describe historical dengue serotype circulation, according to age and geographic location, in a pediatric Indonesian population. Dengue IgG-positive sera, collected from 30 sites across urban Indonesia, were tested by the PRNT to measure the prevalence and concentration of serotype-specific neutralizing antibodies according to subject age and geography.* | Materials and Methods - Study design |
| Setting | 5 | Describe the setting, locations, and relevant dates, including periods of recruitment, exposure, follow-up, and data collection  *Surveillance and sample collection methods were previously described. Briefly, between 30 October 2014 – 27 November 2014, blood samples were collected from 3,210 children aged 1–18 years in 30 urban Indonesian subdistricts, randomly selected from west to east based on the probability proportional to population size. The blood samples were to be tested for dengue IgG by enzyme-linked immunosorbent assay (ELISA). A sub-sample of 780 dengue IgG positive sera was used to estimate the prevalence of serotype-specific neutralizing antibodies by PRNT. The sample size was estimated to provide 95% confidence and a margin error of 5%,; this is accounting for the 30 clusters with a design effect of two and assuming the “worst case” of 50% exposure to any one serotype. The sample was not strictly representative of the dengue IgG positive population as the samples were selected equally from each of the four age groups and, to provide geographical representativeness, from clusters in proportion to dengue IgG seroprevalence rates. This method over-sampled from younger subjects to; 1) increase the number of samples tested from children recently infected with dengue, to provide a record of recent dengue circulation; 2) reduce the number of PRNTs performed on samples from older children, likely to have been infected with many serotypes, which may therefore be impossible to meaningfully interpret.* | Setting – Methods, paragraph 2  Relevant dates/periods of recruitment – Methods, paragraph 2  Exposure – Methods, paragraph 2  Follow-up – (no follow-up in the study) Data collection – Methods, paragraph 2 |
| Participants | 6 | (*a*) Give the eligibility criteria, and the sources and methods of selection of participants  *Surveillance and sample collection methods were previously described. Briefly, between 30 October 2014 – 27 November 2014, blood samples were collected from 3,210 children aged 1–18 years in 30 urban Indonesian subdistricts, randomly selected from west to east based on the probability proportional to population size.* | Methods, paragraph 2 |
| Variables | 7 | Clearly define all outcomes, exposures, predictors, potential confounders, and effect modifiers. Give diagnostic criteria, if applicable  T*he PRNT method was performed based on optimized and validated PRNT50 assay for the detection of neutralizing antibodies to four serotypes of DENV . The parental DENVs of the recombinant CYD vaccine viruses, i.e., DENV-1 strain PUO-359, DENV-2 strain PUO-218, DENV-3 strain PaH881/88, and DENV-4 strain 1228, were used as challenge viruses in the PRNT. The initial source, and the suitability of these four DENV serotypes to be used in dengue neutralization assay have been described elsewhere. Dengue-antibody positive and negative human serum sample controls were obtained from healthy adult donors from Indonesia. The serum controls were used in each assay run, and served to monitor its performance and validity.*  *The neutralization titer (PRNT50) of the test serum sample was defined as the reciprocal of the highest test serum dilution for which the virus infectivity was reduced by 50% when compared with the average plaque count of the challenge virus control, calculated using a four-point linear regression method. Since the lowest starting dilution of serum in the assay was 1:5, the theoretical lower limit of quantitation of the assay was a titer of 10 (reciprocal dilution).* | Materials and Methods – paragraphs 3 and 4 |
| Data sources/ measurement | 8* | For each variable of interest, give sources of data and details of methods of assessment (measurement). Describe comparability of assessment methods if there is more than one group  *The PRNT method was performed based on optimized and validated PRNT50 assay for the detection of neutralizing antibodies to four serotypes of DENV. Each serum sample was heat inactivated at 56°C and assayed in four separate PRNT runs, which corresponded to four different DENV serotype challenge viruses. Vero cells (CCL-81) were obtained from American Type Culture Collection (ATCC). Cells were grown and maintained in Minimum Essential Medium (MEM) (Gibco-Thermo Fisher Scientific, CA, USA), supplemented with 5% heat-inactivated Fetal Bovine Serum (FBS), 2 mM of L-glutamine, and 1% of antibiotic/antimycotic (Gibco-Thermo Fisher Scientific, CA, USA) at 37°C in an atmosphere of 5% CO2. Working banks of Vero cells were prepared in-house, qualified, and confirmed to be free of any microbial, mycoplasma, and viral contaminants. Purified mouse monoclonal antibodies (MAbs) specific to the DENV serotype envelope protein were used as the primary antibodies for virus detection according to the corresponding serotype: anti-DENV-1 (D2-1F1-3), anti-DENV-2 (3H5-1-12), anti-DENV-3 (8A1-2F12), and anti-DENV-4 (1H10-6-7) (Biotem, Le Rivier d’Apprieu, France). Alkaline phosphatase-conjugated goat anti-mouse IgG (Jackson Immunoresearch Laboratories, West Grove, PA, USA) was used as the secondary antibody. The parental DENVs of the recombinant CYD vaccine viruses, i.e., DENV-1 strain PUO-359, DENV-2 strain PUO-218, DENV-3 strain PaH881/88, and DENV-4 strain 1228, were used as challenge viruses in the PRNT. The initial source, and the suitability of these four DENV serotypes to be used in dengue neutralization assay have been described elsewhere. Dengue-antibody positive and negative human serum sample controls were obtained from healthy adult donors from Indonesia. The serum controls were used in each assay run, and served to monitor its performance and validity.*  *The neutralization titer (PRNT50) of the test serum sample was defined as the reciprocal of the highest test serum dilution for which the virus infectivity was reduced by 50% when compared with the average plaque count of the challenge virus control, calculated using a four-point linear regression method. Since the lowest starting dilution of serum in the assay was 1:5, the theoretical lower limit of quantitation of the assay was a titer of 10 (reciprocal dilution).* | Materials and Methods – paragraphs 3 and 4 |
| Bias | 9 | Describe any efforts to address potential sources of bias  *The sample was not strictly representative of the dengue IgG positive population as the samples were selected equally from each of the four age groups and, to provide geographical representativeness, from clusters in proportion to dengue IgG seroprevalence rates. This method over-sampled from younger subjects to; 1) increase the number of samples tested from children recently infected with dengue, to provide a record of recent dengue circulation; 2) reduce the number of PRNTs performed on samples from older children, likely to have been infected with many serotypes, which may therefore be impossible to meaningfully interpret.* | Materials and Methods – paragraph 2 |
| Study size | 10 | Explain how the study size was arrived at  *A sub-sample of 780 dengue IgG positive sera was used to estimate the prevalence of serotype-specific neutralizing antibodies by PRNT. The sample size was estimated to provide 95% confidence and a margin error of 5%; this is accounting for the 30 clusters with a design effect of two and assuming the “worst case” of 50% exposure to any one serotype.* | Materials and Methods – paragraph 2 |
| Quantitative variables | 11 | Explain how quantitative variables were handled in the analyses. If applicable, describe which groupings were chosen and why  *In this cross-sectional study, data from a pediatric dengue seroprevalence study was used to describe historical dengue serotype circulation, according to* ***age and geographic*** *location, in a pediatric Indonesian population. Dengue IgG-positive sera, collected from 30 sites across urban Indonesia, were tested by the PRNT to measure the prevalence and concentration of serotype-specific neutralizing antibodies according to subject* ***age and geography.*** | Materials and Methods – paragraphs 1 and 2 |
| Statistical methods | 12 | (*a*) Describe all statistical methods, including those used to control for confounding  *The study population mean age was calculated and geographic distribution described. Dengue serotype specific age and geographic distribution was described according to the following algorithm; categorizing samples as naïve (no previous dengue infection), monotypic (infection with one serotype), or multitypic (>1 serotype):*   - *Naïve: antibody titers <10 for the four serotypes* - *Monotypic: antibody titers >10 (1/dil) to only one serotype* ***or*** *titers ≥ 10 for different serotypes with a high titer (>80 (1/dil)) and for a single predominant serotype (> 5 times higher than other titers)* - *Multitypic: antibody titers ≥10 (1/dil) for different serotypes without a single predominant titer.*   *PRNT profile prevalence and their 95% confidence interval (95% CI) were calculated, the clusters results were aggregated at province level and a map was generated using QGIS 2.16.2 “Nødebo”.*  *The mean PRNT titer, GMT (Geometric Mean Titer), per age group and dengue serotype were calculated with their 95% CI. To calculate the GMT, samples with an antibody titer T <10 (1/dil) were given the value 5 and the mean titer was calculated using the equation:*  *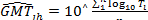*  *Where 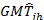 is the mean titer for the dengue serotype h of the age group j, 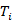 is the PRNT titer of the subjects i and n the number of subjects with a PRNT titer in the age group j for the serotype h.*  *All statistical analyses were performed using Excel 2013.* | Methods, Statistical analysis, paragraphs 1–3 |
|  |  | (*b*) Describe any methods used to examine subgroups and interactions | NA, no subgroups. |
|  |  | (*c*) Explain how missing data were addressed | NA |
|  |  | (*d*) If applicable, describe analytical methods taking account of sampling strategy  *Dengue serotype specific age and geographic distribution was described according to the following algorithm; categorizing samples as naïve (no previous dengue infection), monotypic (infection with one serotype), or multitypic (>1 serotype):*   - *Naïve: antibody titers <10 for the four serotypes* - *Monotypic: antibody titers >10 (1/dil) to only one serotype* ***or*** *titers ≥ 10 for different serotypes with a high titer (>80 (1/dil)) and for a single predominant serotype (> 5 times higher than other titers)* - *Multitypic: antibody titers ≥10 (1/dil) for different serotypes without a single predominant titer.* | Methods, Statistical analysis, paragraphs 1–3 |
|  |  | (*e*) Describe any sensitivity analyses | NA |
| Results | | |  |
| Participants | 13* | (a) Report numbers of individuals at each stage of study—eg numbers potentially eligible, examined for eligibility, confirmed eligible, included in the study, completing follow-up, and analysed  *Blood samples were collected from 3,210 children aged 1–18 years in 30 urban Indonesian subdistricts, randomly selected from west to east. From a sub-sample of 780 dengue IgG positive sera, PRNT50 results were obtained from 776 participants, equally sampled from each age group (1–4, 5–9, 10–14 and 15–18 years old). In the youngest, 1–4 years old group, four serum samples were of insufficient quantity to be tested. The mean age was 9.6 years old (95% CI [9.3–10.0]. The 30 clusters were represented with 14–39 samples per cluster. Of these, 765 (98.6%) neutralized one or more dengue serotypes at a threshold of >10 (1/dil), a proportion which varied by age: 95.3% in the 1–4 years old, 99.5% in the 5–9 years old, 99.5% in the 10–14 years old and 100% in the 15–18 years old.* | Results, Description of sample set, paragraph 1 |
|  |  | (b) Give reasons for non-participation at each stage  *In the youngest, 1–4 years old group, four serum samples were of insufficient quantity to be tested.* | Results, Description of sample set, paragraph 1 |
|  |  | (c) Consider use of a flow diagram | NA |
| Descriptive data | 14* | (a) Give characteristics of study participants (eg demographic, clinical, social) and information on exposures and potential confounders  *Blood samples were collected from 3,210 children aged 1–18 years in 30 urban Indonesian subdistricts, randomly selected from west to east. From a sub-sample of 780 dengue IgG positive sera, PRNT50 results were obtained from 776 participants, equally sampled from each age group (1–4, 5–9, 10–14 and 15–18 years old).* | Results, paragraph 1 |
|  |  | (b) Indicate number of participants with missing data for each variable of interest | NA |
| Outcome data | 15* | Report numbers of outcome events or summary measures |  |
| Main results | 16 | (*a*) Give unadjusted estimates and, if applicable, confounder-adjusted estimates and their precision (eg, 95% confidence interval). Make clear which confounders were adjusted for and why they were included  *PRNT50 (95% confidence intervals, PRNT profile distribution stratified by age and geographic level* | Results, paragraphs 1, 2, 3 and 4 |
|  |  | (*b*) Report category boundaries when continuous variables were categorized | NA |
|  |  | (*c*) If relevant, consider translating estimates of relative risk into absolute risk for a meaningful time period | NA |
| Other analyses | 17 | Report other analyses done—eg analyses of subgroups and interactions, and sensitivity analyses | NA |
| Discussion | | |  |
| Key results | 18 | Summarise key results with reference to study objectives  *We conducted a dengue seroprevalence study which identified serological evidence for the circulation of all four dengue serotypes across urban areas of Indonesia, in children who were exposed to infection from 1996 to 2013. The proportion of children with exposure to >1 serotype increased with age, and children were more likely to have been infected with DENV-2, DENV-1 and DENV-3 than DENV-4. Nonetheless, these results show that all four serotypes have been widely circulating in most of Indonesia, as is common in hyper-endemic countries. This study generated data on serotype-specific prevalence in areas where little or no data were previously available.* | Discussion, paragraph 1 |
| Limitations | 19 | Discuss limitations of the study, taking into account sources of potential bias or imprecision. Discuss both direction and magnitude of any potential bias  *Samples were collected from suspected cases and therefore suffer a potential selection bias towards serotypes associated with more symptomatic/severe cases.*  *There are several limitations to our study. Sera collected during the convalescent phase represent infection history in the population, but are limited by the sensitivity and specificity of the serological methods used to quantify antibodies. We had the benefit of analyzing samples in this study by PRNT; however interpretation of data can be confused by heterotypic cross-neutralization between serotypes. For this reason, we did not interpret the serotype distributions of multitypic infections. Only samples positive for dengue IgG in ELISA screening assay were selected to undergo PRNT, therefore these may not be fully representative of dengue positive sera. Our sample collection was also limited to urban areas and subjects consenting to the study which may have introduced additional bias.* | Discussion, paragraphs 3 and 5 |
| Interpretation | 20 | Give a cautious overall interpretation of results considering objectives, limitations, multiplicity of analyses, results from similar studies, and other relevant evidence  *In summary, available dengue serotype data collated from 1994 to 2012 (n=596) from all over Indonesia, confirm the concomitant presence of the four DENV serotypes. Samples were collected from suspected cases and therefore suffer a potential selection bias towards serotypes associated with more symptomatic/severe cases. The serological data we report here indicate a consistent pattern of distribution of serotypes, a finding which may indicate that the cases captured within these surveillance studies is broadly reflective of the DENV serotype circulation in the country.* | Discussion, paragraph 3 |
| Generalisability | 21 | Discuss the generalisability (external validity) of the study results  *PRNT enables the interrogation of samples according to their exposure history. In this study, it was remarkable to observe that in this pediatric population more than half (50.9%) had already been exposed to >1 dengue serotype, a proportion which increased with age. This statistic is important because it demonstrates early and intense transmission in Indonesia; and we know that second infections have been described as more likely to be symptomatic, severe and hemorrhagic. Individuals of an age likely to have received one natural exposure, but before their second, may represent an attractive target for dengue vaccination programs. The observed GMT increase with age is most likely explained by continuous re-exposure to wild type DENVs over time, further boosting antibody levels. Serological data collected in 1995, from Yogyakarta, in the central area of Java island, support a conclusion that four DENVs have been circulating in Indonesia for many years .* | Discussion, paragraph 4 |
| Other information | | |  |
| Funding | 22 | Give the source of funding and the role of the funders for the present study and, if applicable, for the original study on which the present article is based | Included in supplemental material |

*Give information separately for exposed and unexposed groups.

**Note:** An Explanation and Elaboration article discusses each checklist item and gives methodological background and published examples of transparent reporting. The STROBE checklist is best used in conjunction with this article (freely available on the Web sites of PLoS Medicine at http://www.plosmedicine.org/, Annals of Internal Medicine at http://www.annals.org/, and Epidemiology at http://www.epidem.com/). Information on the STROBE Initiative is available at www.strobe-statement.org.
