# Supplementary material for: Dengue virus serotype distribution based on serological evidence in pediatric urban population in Indonesia
Source: PLoS Negl Trop Dis. 2018 Jun 28;12(6):e0006616. doi: 10.1371/journal.pntd.0006616 (PMC6040755; doi:10.1371/journal.pntd.0006616)
Supplement: S1 Table — (DOCX) [file pntd.0006616.s002.docx]

**Supplementary Table S1. Mean age (years), sample size and dengue serotype-specific antibody prevalence (naïve, monotypic for each serotype, or multitypic) per province.**

| **Province** | **Mean age** | **N cluster** | **n subject** | **Naïve** | **DENV-1** | **DENV-2** | **DENV-3** | **DENV-4** | **Multi** |
| --- | --- | --- | --- | --- | --- | --- | --- | --- | --- |
| Nanggroe Aceh Darussalam | 10.8 | 1 | 23 | 0.0% | 17.4% | 0.0% | 13.0% | 17.4% | 52.2% |
| Sumatera Utara | 9.2 | 1 | 30 | 0.0% | 6.7% | 36.7% | 3.3% | 13.3% | 40.0% |
| Sumatera Barat | 9.2 | 1 | 28 | 0.0% | 14.3% | 25.0% | 25.0% | 3.6% | 32.1% |
| Jambi | 9.2 | 1 | 28 | 0.0% | 3.6% | 7.1% | 17.9% | 10.7% | 60.7% |
| Lampung | 10.9 | 1 | 15 | 6.7% | 20.0% | 20.0% | 6.7% | 6.7% | 40.0% |
| Banten | 8.2 | 2 | 62 | 0.0% | 8.1% | 11.3% | 16.1% | 1.6% | 62.9% |
| Dki Jakarta | 9.8 | 3 | 78 | 3.8% | 16.7% | 14.1% | 16.7% | 5.1% | 43.6% |
| Jawa Barat | 9.7 | 7 | 183 | 3.3% | 13.7% | 21.3% | 12.0% | 2.7% | 47.0% |
| Jawa Tengah | 9.8 | 4 | 112 | 0.9% | 8.0% | 15.2% | 11.6% | 5.4% | 58.9% |
| Jawa Timur | 10.0 | 5 | 117 | 0.0% | 21.4% | 12.0% | 14.5% | 0.9% | 51.3% |
| Bali | 10.1 | 1 | 24 | 0.0% | 12.5% | 12.5% | 12.5% | 4.2% | 58.3% |
| Kalimantan Timur | 10.1 | 1 | 24 | 0.0% | 16.7% | 25.0% | 12.5% | 4.2% | 41.7% |
| Sulawesi Selatan | 10.4 | 1 | 16 | 0.0% | 12.5% | 43.8% | 12.5% | 0.0% | 31.3% |
| Sulawesi Tenggara | 8.6 | 1 | 36 | 0.0% | 5.6% | 11.1% | 13.9% | 0.0% | 69.4% |
| **Total** | **9.6** | **30** | **776** | **1.4%** | **13.1%** | **16.9%** | **13.5%** | **4.1%** | **50.9%** |
