# Supplementary material for: Dengue virus serotype distribution based on serological evidence in pediatric urban population in Indonesia
Source: PLoS Negl Trop Dis. 2018 Jun 28;12(6):e0006616. doi: 10.1371/journal.pntd.0006616 (PMC6040755; doi:10.1371/journal.pntd.0006616)
Supplement: S2 Table — (DOCX) [file pntd.0006616.s003.docx]

**Supplementary Table S2.** IgG seroprevalance per cluster and age group; and resulting number of samples subjected to PRNT_50_

| Age group | 1 – 4 years | | | 5 – 9 years | | | 10 – 14 years | | | 15 – 18 years | | |
| --- | --- | --- | --- | --- | --- | --- | --- | --- | --- | --- | --- | --- |
| Site | N IgG | IgG seroprevalence % | N (PRNT) | N IgG | IgG seroprevalence % | N (PRNT) | N IgG | IgG seroprevalence % | N (PRNT) | N IgG | IgG seroprevalence % | N (PRNT) |
| 1 | 21 | 23% | 4 | 31 | 57% | 6 | 27 | 83% | 6 | 27 | 85% | 7 |
| 2 | 22 | 45% | 9 | 30 | 72% | 8 | 28 | 86% | 6 | 27 | 93% | 7 |
| 3 | 23 | 32% | 7 | 29 | 83% | 9 | 29 | 79% | 6 | 25 | 88% | 6 |
| 4 | 25 | 36% | 8 | 28 | 72% | 7 | 28 | 93% | 7 | 26 | 96% | 7 |
| 5 | 23 | 10% | 2 | 29 | 38% | 4 | 31 | 59% | 5 | 22 | 69% | 4 |
| 6 | 25 | 82% | 17 | 25 | 89% | 8 | 31 | 86% | 7 | 26 | 93% | 7 |
| 7 | 20 | 35% | 6 | 28 | 59% | 6 | 27 | 72% | 5 | 26 | 81% | 6 |
| 8 | 21 | 41% | 8 | 28 | 71% | 7 | 31 | 89% | 7 | 25 | 96% | 7 |
| 9 | 22 | 50% | 9 | 29 | 61% | 6 | 29 | 79% | 6 | 27 | 93% | 7 |
| 10 | 22 | 18% | 3 | 30 | 52% | 5 | 29 | 93% | 7 | 26 | 93% | 7 |
| 11 | 22 | 73% | 14 | 29 | 69% | 7 | 30 | 76% | 6 | 26 | 96% | 7 |
| 12 | 22 | 36% | 7 | 29 | 52% | 5 | 31 | 76% | 6 | 25 | 85% | 6 |
| 13 | 28 | 18% | 7 | 27 | 69% | 7 | 31 | 93% | 8 | 21 | 100% | 6 |
| 14 | 22 | 27% | 5 | 27 | 86% | 8 | 30 | 97% | 8 | 27 | 93% | 7 |
| 15 | 22 | 35% | 7 | 29 | 83% | 8 | 29 | 100% | 8 | 27 | 97% | 7 |
| 16 | 22 | 32% | 6 | 30 | 48% | 5 | 29 | 97% | 7 | 26 | 89% | 7 |
| 17 | 22 | 14% | 3 | 29 | 41% | 4 | 29 | 52% | 4 | 27 | 67% | 5 |
| 18 | 22 | 0% | 0 | 29 | 52% | 6 | 29 | 77% | 6 | 27 | 96% | 7 |
| 19 | 23 | 23% | 4 | 31 | 69% | 8 | 29 | 100% | 8 | 24 | 100% | 7 |
| 20 | 22 | 55% | 10 | 29 | 83% | 8 | 29 | 97% | 8 | 27 | 100% | 8 |
| 21 | 22 | 41% | 8 | 29 | 90% | 9 | 30 | 93% | 8 | 26 | 100% | 7 |
| 22 | 22 | 23% | 4 | 29 | 29% | 3 | 29 | 48% | 4 | 27 | 37% | 3 |
| 23 | 22 | 9% | 2 | 30 | 50% | 5 | 29 | 86% | 7 | 25 | 96% | 7 |
| 24 | 22 | 18% | 3 | 30 | 76% | 8 | 29 | 100% | 8 | 26 | 96% | 7 |
| 25 | 23 | 14% | 3 | 28 | 55% | 6 | 30 | 78% | 6 | 24 | 96% | 7 |
| 26 | 22 | 64% | 12 | 29 | 83% | 8 | 29 | 90% | 7 | 27 | 96% | 7 |
| 27 | 22 | 27% | 5 | 29 | 72% | 7 | 30 | 79% | 6 | 26 | 85% | 6 |
| 28 | 22 | 36% | 7 | 29 | 41% | 4 | 29 | 76% | 6 | 27 | 93% | 7 |
| 29 | 23 | 18% | 3 | 22 | 55% | 4 | 35 | 38% | 4 | 27 | 70% | 5 |
| 30 | 21 | 68% | 12 | 30 | 83% | 9 | 30 | 97% | 8 | 26 | 100% | 7 |
